# Bendamustine and rituximab as frontline therapy in extranodal marginal zone lymphoma: a single-institution experience

**DOI:** 10.32604/or.2024.046885

**Published:** 2024-05-23

**Authors:** CONSTANTINE N. LOGOTHETIS, NATHAN P. HORVAT, TONY KURIAN, CELESTE BELLO, JULIO CHAVEZ, LEIDY ISENALUMHE, BIJAL SHAH, LUBOMIR SOKOL, HAYDER SAEED, JAVIER PINILLA, SAMEH GABALLA

**Affiliations:** 1 Department of Internal Medicine, University of South Florida, Tampa, FL, 33602, USA; 2 College of Medicine, University of South Florida Morsani, Tampa, FL, 33602, USA; 3 H. Lee Moffitt Cancer Center and Research Institute, Tampa, FL, 33612, USA

**Keywords:** Extranodal marginal zone lymphoma, Bendamustine, Rituximab, Front-line therapy

## Abstract

Extranodal marginal zone lymphoma (EMZL) encompasses 70% of cases of marginal zone lymphoma. Frontline bendamustine and rituximab (BR) were derived from trials involving other indolent non-Hodgkin’s lymphomas. Only one trial has evaluated frontline BR prospectively in EMZL. This retrospective study reports outcomes among EMZL patients receiving frontline BR. Twenty-five patients were included with a median age of 69 years (40–81). Five (20.0%) patients had stage I/II disease, and 20 (80.0%) had stage III/IV disease. The median number of cycles was 6.0 (3.0–6.0). Maintenance rituximab was administered to 10 (41.7%) individuals. Overall response rate (ORR) was 100.0% (60.0% complete response, 40.0% partial response). Medians of overall survival and progression-free survival were not reached. The estimated 2-year progression-free survival was 85.2% and overall survival was 100.0%. Four (16.6%) patients had infections related to treatment; 3 (12.0%) transformed to diffuse large B-cell lymphoma; 5 (20.8%) had a relapse or progression of EMZL; and 3 (12.0%) died unrelated to BR. BR is an efficacious and well-tolerated front-line regimen for EMZL with response data consistent with existing literature.

## Introduction

Marginal-zone lymphoma (MZL) is the second most common indolent B-cell non-Hodgkin’s lymphoma after follicular lymphoma and represents a group of different lymphomas that arise from the post-germinal center marginal-zone B-cells. MZL subtypes include splenic, nodal, and extranodal (gastric and nongastric). Extranodal MZL encompasses roughly 70% of the MZL diagnoses [1] and arises from different epithelial cells, including gastric (30%), ocular adnexa (12%), skin (10%), lung (9%), and salivary glands (7%) [2].

Many patients with EMZL can be observed initially, but therapy is indicated for symptomatic patients or those with bulky disease. While patients with early stage EMZL including gastric EMZL are commonly treated with radiation therapy, others with more advanced disease will need systemic therapy. However, there is no consensus on the optimal frontline systemic therapy for patients with EMZL, and most of the data are extrapolated from retrospective series or from prospective indolent-lymphoma trials that included a subset of MZL patients. Common systemic regimens include single-agent rituximab [3–5], rituximab with bendamustine (BR) [6], lenalidomide with rituximab [7,8], R-CVP [rituximab, cyclophosphamide, vincristine, and prednisolone] [6,9], and R-CHOP [rituximab, cyclophosphamide, doxorubicin, vincristine, and prednisone] [10,11]. The International Extranodal Lymphoma Study Group 19 is the only randomized phase III trial that exclusively enrolled patients with EMZL and randomized patients to chlorambucil plus rituximab *vs*. chlorambucil *vs*. rituximab arms and demonstrated superior event-free survival and progression-free survival (PFS) with the combination compared to either of thew monotherapy arms. However, there was no statistical difference in the overall survival (OS), and the combination regimen is rarely used in the United States [12]. BR is commonly used in the United States as a frontline treatment option in EMZL; however, the data supporting the use of BR as initial therapy for EMZL remain limited [13]. Two randomized control trials have evaluated BR *vs*. R-CVP or R-CHOP among indolent B-cell lymphomas more broadly but primarily consisted of patients with follicular lymphoma [6]. Lastly, the multicenter phase 2 MALT2008-01 study evaluated BR in treatment-naive EMZL and found superior responses with 100% of patients responding, 98% of them with a complete response (CR) or unconfirmed response with an associated 7-year event-free survival of 88%. While the MALT2008-01 study prospectively evaluated BR in EMZL patients with EMZL, roughly 50% of the study patients had gastric EMZL [14].

Because of the paucity of available literature, further studies assessing the safety and efficacy of BR as initial treatment for newly diagnosed EMZL are warranted. Retrospective studies provide real-world data without the stringent inclusion and exclusion criteria of randomized controlled trials. This single-institution, retrospective, real-world experience seeks to explore the safety and efficacy of BR as frontline therapy for untreated nongastric EMZL.

## Materials and Methods

This retrospective study consisted of patients from a single large academic cancer institution. The institutional registry was queried for patients older than 18 years with EMZL who received first-line BR between 2011 and 2020. All patients 18 years or older with primary treatment-naive EMZL receiving frontline BR were included. Twenty-five patients were included based upon the above inclusion criteria. This study was approved by the University of South Florida institutional review board, which includes an ethical scientific review under IRB number Pro0061722. Due to the retrospective nature of the study, informed consent was waived under the IRB approval. Demographic data, clinical characteristics, and outcome data were extracted and recorded. Staging of EMZL was based on Ann Arbor Staging [15]. Staging was evaluated using fluorodeoxyglucose positron emission tomography, using the Lugano classification [16]. Median follow-up time was calculated from the start date of BR to the date of the last follow-up for living patients. Responses were assessed by computed tomography using the 2007 Cheson criteria [17]. SPSS software was used to perform statistical analysis. PFS and OS were estimated using the Kaplan-Meier analysis and log-rank test.

## Results

### Basic demographics

Patient characteristics are summarized in Table 1. Twenty-five patients were included in the study analysis. The median age was 69 years (40–81). Five patients (20.0%) had stage I/II disease while 20 (80.0%) patients had stage III/IV disease. The mucosa-associated lymphoid tissue International Prognostic Index (MALT-IPI) was available for 19 patients as follows: low risk (10.5%), intermediate risk (47.5%), and high risk (42.1%). ECOG performance status was less than 2 for all patients. Twenty-four individuals had available data regarding *Helicobacter pylori* (*H. pylori*) infections. One patient (4%) patient had a biopsy-proven *H. pylori* infection that was adequately treated. History of autoimmune conditions was available for 21 patients. Three (14%) patients had a history of autoimmune diseases, including primary hypothyroidism, sarcoidosis, and Sjögren’s syndrome. The most common sites of EMZL involvement were lungs (N = 7, 28.0%), skin and soft tissue (N = 5, 20.0%), thyroid (N = 5, 20.0%), urinary (N = 3, 12.0%), gastrointestinal (nongastric) (N = 2, 8.0%), multiple sites (N = 2, 8.0%), and ocular (N = 1, 4.0%).

**Table 1:** Patient demographics, disease parameters, and treatment-related outcomes

	Patient cohort (N = 25)
Characteristics	Median (Range) Count (%)
Age at diagnosis in years	69 (40–81)
Gender:	
Male	15 (60)
Female	10 (40)
Race:	
White:	20 (80)
Asian:	1 (4)
Other:	4 (16)
Stage at diagnosis:	
Stage I or II:	5 (20)
Stage III or IV:	20 (80)
MALT-IPI (N = 19):	
Low:	2 (10.5)
Intermediate:	9 (47.5)
High:	8 (42.1)
Presences of B-symptoms (N = 23):	
No B-Symptoms	19 (82.6)
Presence of B-symptoms	4 (17.4)
Bone marrow involvement (N = 23):	
Bone marrow involvement:	19 (82.6)
No bone marrow involvement:	4 (17.4)
Elevated laboratory values:	
LDH U/L (N = 19):	5 (26.3)
Beta-2 microglobulin mg (N = 17):	13 (76.5)
Monoclonal IgM mg/dL (N = 23):	6 (26.1)
Hemoglobin < 12.0 g/dL (N = 19)	6 (31.6)
Location of extra-nodal involvement (N = 25):	
Lungs	7 (28)
Skin	5 (20)
Thyroid	5 (20)
Urinary	3 (12)
Gastrointestinal (Non-gastric)	2 (8)
Multiple sites	2 (8)
Ocular	1 (4)
Treatments:	
Median number of BR cycles (Range):	6 (3–6)
Time to initiation of BR in months: Maintenance rituximab (N = 24):	2.4 (0.3–25.5) 10 (41)
Treatment response (N = 25):	
Overall response:	25 (100)
Complete response:	15 (60)
Partial response:	10 (40)
Survival data:	
2- year estimated progression free survival:	85.2%
2-year estimated overall survival:	100.0%
relapse/progression (N = 25):	5 (20%)
Time to relapse/progression (Months)	4.1–47.8
Treatment complications:	
Infections (N = 24):	4 (16.6%)
Shingles	1 (4.2%)
Skin/soft tissue infection	1 (4.2%)
Pneumonia	1 (4.2%)
Herpes simplex virus reactivation	1 (4.2%)
Transformation to DLBCL	
Transformation to DLBCL (N = 25):	3 (12.0%)
Time to transformation in months:	7.8–25.8
Follow up data:	
Median follow up in months:	27 (3.6–104.9)
Alive:	22 (88.0%)
Deceased:	3 (12.0%)

### Treatment descriptions

The median number of BR cycles was 6 (3–6). The median time to initiate therapy from the time of diagnosis was 2.4 months (0.3–25.5). Maintenance rituximab was administered to 10 (41.7%) patients with a median duration of 24 (18–24) months.

### Efficacy and safety analysis

**Figure 1:**
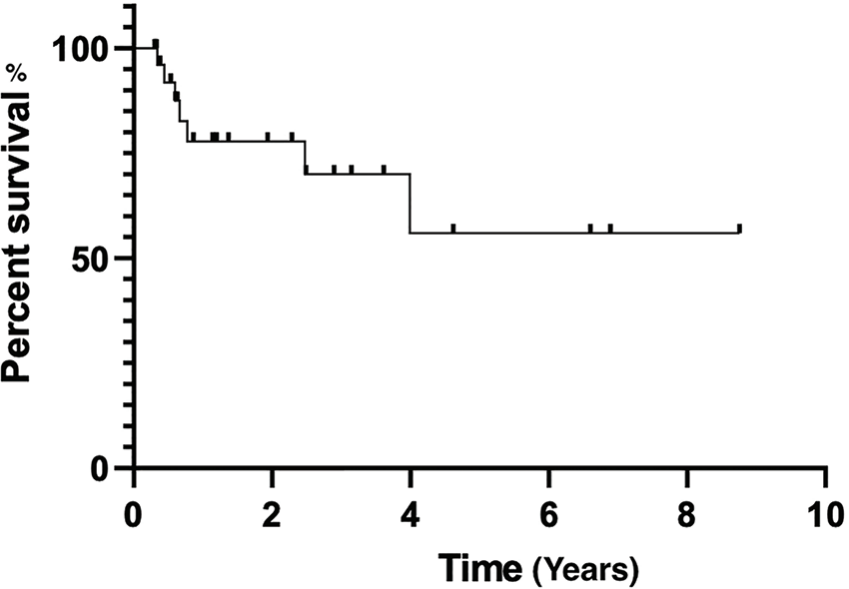
Progression-free survival estimated by Kaplan-Meier curve.

**Figure 2:**
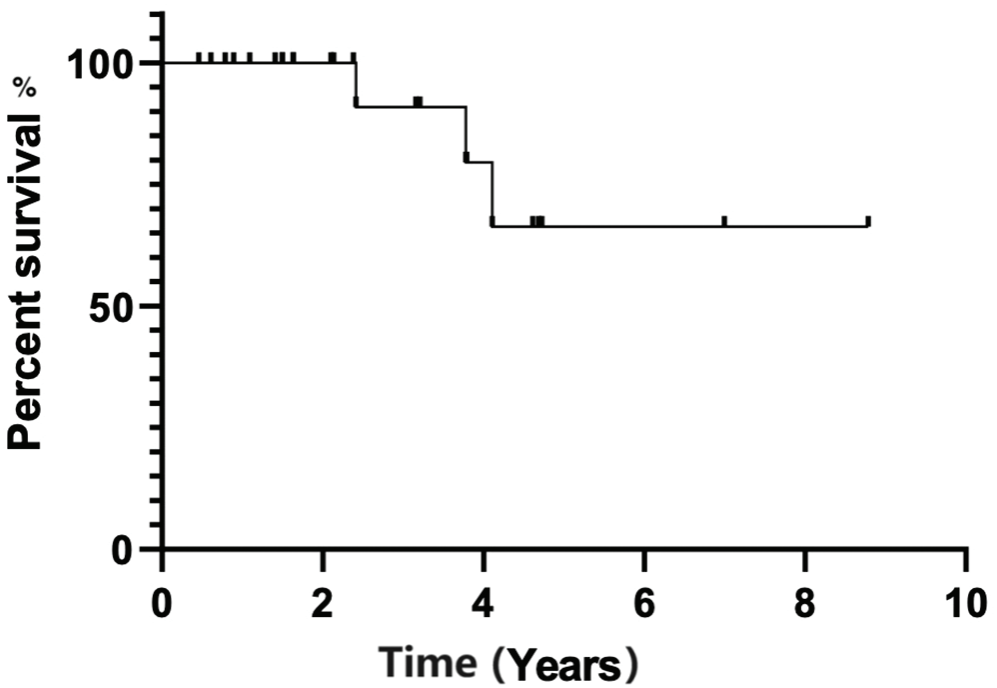
Overall survival estimated by Kaplan-Meier curve.

The regimen of BR elicited an overall response (OR) of 100.0% (60.0% CR, 40.0% partial response). With a median follow-up of 27 (3.6–104.9) months, the median OS and PFS were not reached yet. The estimated two-year PFS was 85.2%, and two-year OS was 100.0% (Figs. 1, 2).

**Figure 3:**
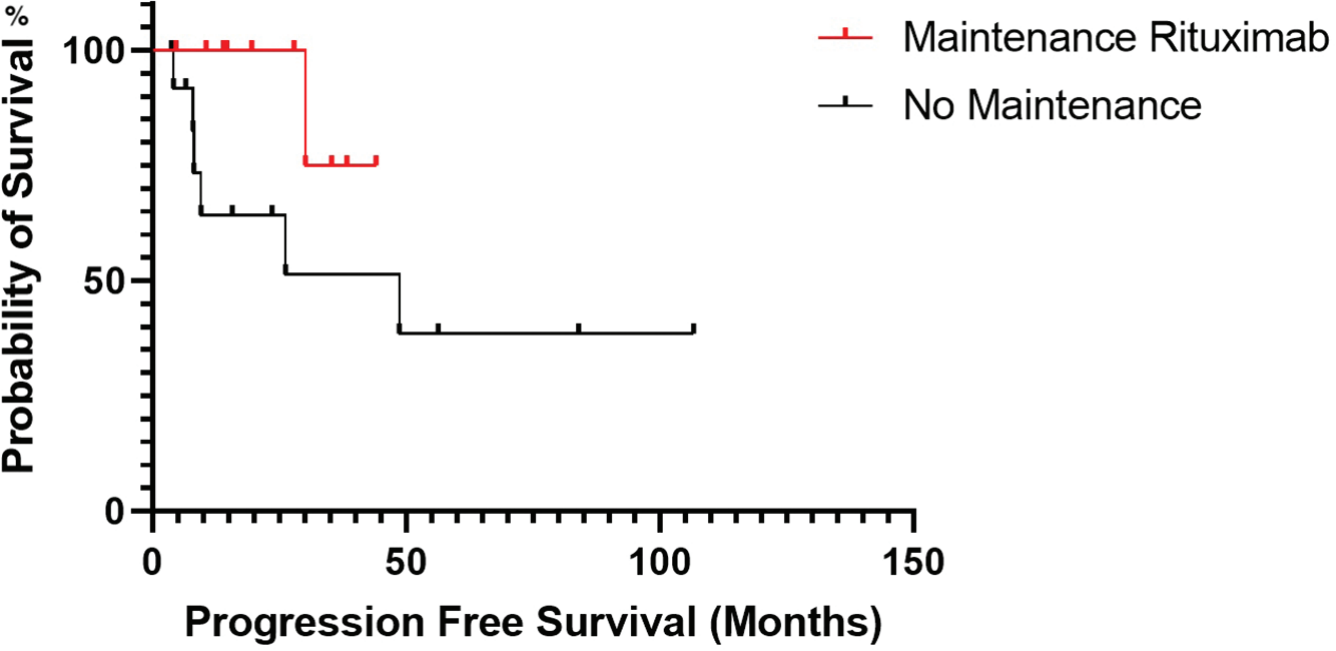
Progression-free survival estimated by Kaplan-Meier curve for patients treated with rituximab maintenance *vs*. no rituximab maintenance.

**Figure 4:**
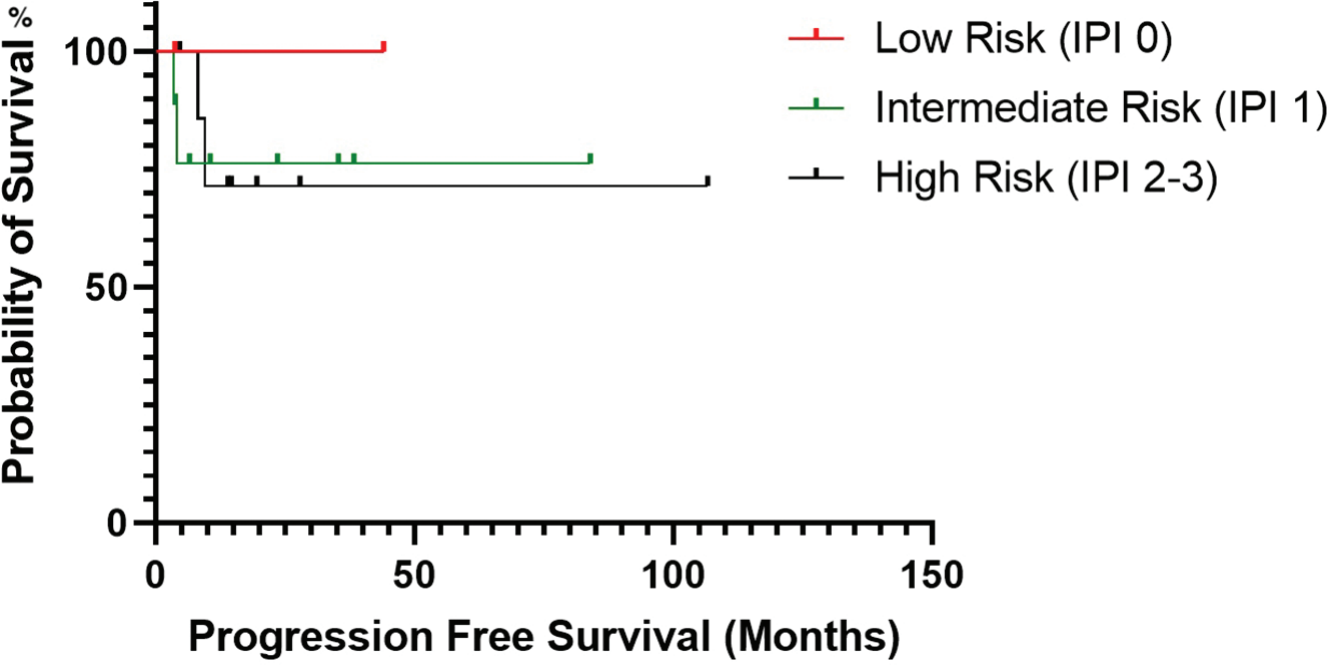
Progression-free survival estimated by Kaplan-Meier curve based on the patient’s mucosa-associated lymphoid tissue International Prognostic Index score.

There was no statistical significance for PFS between patients who received maintenance rituximab *vs*. those who did not receive maintenance therapy (*p*-value, 0.109) (Fig. 3). Additionally, there was no statistical significance in PFS (*p*-value, 0.827) between patients when stratified by mucosa-associated lymphoid tissue International Prognostic Index score (Fig. 4).

Four (16.6%) patients developed treatment-related infectious complications, including shingles, pneumonia, skin infection, and herpes simplex virus reactivation. Ten patients had data regarding complications during maintenance rituximab, and only 1 (10%) patient had an infectious complication, in the form of a urinary-tract infection. Three (12.0%) patients transformed to diffuse large B-cell lymphoma (DLBCL) ranging from 7.8 to 25.8 months after initiating BR and were subsequently treated with R-CHOP. None of the transformed patients developed triple-hit rearrangement in MYC, BCL-2, or BCL-6. The best responses to R-CHOP were progressive disease, partial response, and complete remission in the three respective patients. No one underwent stem cell transplantation, including patients with transformed disease. None of the patients developed secondary malignancies during the follow-up. Death occurred in 3 (12.0%) patients. None of the deaths were therapy-related. One patient likely died from progressive DLBCL. Another patient died from amyotrophic lateral sclerosis. The cause of death in the final patient is unknown. Five (20.8%) patients had relapse/progression of EMZL ranging from 4.1 to 47.8 months after treatment.

## Discussion

We retrospectively analyzed the safety and efficacy of BR in treatment of naive EMZL at a single institution and demonstrated that BR was highly efficacious (OR 100%, CR 60%) with a reasonable safety profile. EMZL represents the majority (approximately 70%) of MZL cases and because of a lack of randomized trials exclusive to MZL, there is no consensus on a preferred first-line regimen. In the United States, BR has become the preferred regimen for newly diagnosed EMZL despite little prospective data.

The literature supporting the use of BR for untreated EMZL is derived mainly from indolent-lymphoma trials that enrolled mostly follicular lymphoma patients, with a subset of MZL cases. This included two randomized trials, both with a relatively small MZL patient population: The Study Group for Indolent Lymphoma and the BRIGHT study. The Study Group for Indolent Lymphoma, a noninferiority trial, compared BR to R-CHOP and included 67 patients with MZL patients (37 patients randomized to BR *vs*. 30 patients to R-CHOP), but the percentage of patients with the EMZL subtype was unavailable. There was no statistical difference in PFS between the BR arm and the R-CHOP arm (57 *vs*. 47 months; *p*-value = 0.32). Additionally, the CR rate for the BR arm was 40% *vs*. 30% in the R-CHOP arm [18,19]. The BRIGHT study included only 46 patients with MZL, but again the proportion of patients with EMZL was not provided. Twenty-eight patients were randomized to the BR arm, and 18 patients were randomized to either R-CHOP or R-CVP [rituximab with cyclophosphamide, vincristine, and prednisone]. In this study, the OR was 92% with a CR of 20% among MZL patients [6]. The small number of patients with MZL in both studies may explain the differences in CR rates. The only prospective data available of the efficacy of BR as initial treatment for MZL (specifically MALT) were reported by Salar et al. in a multicenter phase 2 study. The study had 57 evaluable patients with MALT lymphoma and reported an OR of 100% with a high CR of 98%. This CR was much higher than our study (98% *vs*. 60%) and is possibly explained by the fact that one third of the patients in the Salar et al. study had gastric MALT, which has more favorable outcomes than non-gastric EMZL, and that two thirds of patients had early stage I/II disease [14]. Our study did not include any patients with gastric MALT, and only 20% of patients had stage 1 or 2 disease.

Because of the lack of prospective randomized studies investigating the role of BR in treatment-naive MZL, the best available data has been retrospective studies exploring the role of BR in treating MZL. A single-center retrospective analysis included all subtypes of MZL treated with BR, unlike our study which only focused on EMZL. Out of the 65 patients in the study by Morigi et al., 28 (43%) patients had EMZL, and treatment with BR led to an OR of 89.3% and CR of 67.9%, which is in line with our current analysis. There were no treatment-related deaths [19]. The largest retrospective analysis to date was an international consortium study which addressed the utility of BR in untreated EMZL. This study included 237 patients from 20 institutions, including Moffitt Cancer Center (MCC). The OR was 93.2% with a CR of 81%. Our current study included patients who did not overlap with the consortium study, and we again noted a similar OR of 100%. However, the CR in the current study was lower than in the consortium study (60% *vs*. 81%), explained by the retrospective nature of the analysis and the difference in sample size. We did not find a difference in PFS between patients who received maintenance rituximab *vs*. those who did not; however, the consortium study found a PFS advantage (but not OS) with maintenance rituximab likely because of the larger sample size. Taken together, both studies confirmed that BR is highly effective in treating EMZL as a frontline regimen [20]. Both studies demonstrated that BR was safe, except for a small increased rate of infections, particularly viral related (herpes simplex virus or varicella zoster virus). We recommend varicella zoster prophylaxis with acyclovir or valacyclovir for patients undergoing BR at MCC.

Our findings provide a unique perspective on treating EMZL with BR which is a highly effective initial regimen for untreated EMZL. After a median follow-up time of 27.0 months, the median OS and PFS were not reached. The estimated PFS was 85.2% and an OS was 100%. Additionally, patients experienced an OR of 100%, with 60% of patients achieving CR. These findings are generally equal in standing with other published retrospective studies as previously outlined.

There are several limitations to this study. This study is retrospective in nature with inherent biases. Additionally, although this report includes one of the largest patient cohorts evaluating BR among patients with EMZL, the sample size remains limited. Lastly, the follow-up duration was relatively short (27 months) for such an indolent disease. Despite these limitations, our study provides a unique perspective on using BR for treating naive EMZL, particularly given the limited published data available, and our study provides additional evidence supporting its utility. Additionally, all pathology slides were examined at MCC, allowing for uniform, expert review and diagnosis of EMZL at a single institution. Ideally, future randomized trials can further define the role of BR in treating naive MZL in patients compared to other regimens, including newer BTK inhibitors which are currently approved for relapsed MZL.

## Conclusion

Given the scarcity of prospective studies using frontline BR in EMZL, our study provides real-world evidence that BR is a safe and effective therapeutic option for these patients. The response rates and survival outcomes were consistent with other retrospective studies. These responses were durable when compared to single-agent rituximab in the literature although with relatively higher infection rates. The data in this study provides a more generalizable understanding of using BR in EMZL, upholding its utility in the frontline setting.

## Data Availability

The is a retrospective cohort study. Data is available upon request due to privacy and ethical restrictions.
